# Foreign Correspondence

**Published:** 1890-06

**Authors:** William C. Grayston

**Affiliations:** Scarborough, England


### Foreign Correspondence.

To the Editor:

*Copper Amalgam.*—I observe from a perusal of dental journals that the subject of copper amalgam has of late years received a great deal of attention from dentists in America; that many instructive articles have been written concerning it; and that several new or improved makes of this material are now on the market. For the last six years I have used a considerable quantity of copper amalgam, known here as Sullivan's cement, and which has for very many years been extensively used in this country. During this period I have filled quite two-thirds of all cavities in molar teeth with it, and I have therefore thought that some account of my experience with it may be useful as a slight addition to what has already been written on the subject, although much that I have to state has been observed by others and frequently discussed.

Sullivan's cement has been described as a dirty compound, certain to blacken teeth, and fit to be used only in the mouths of those whose uncleanly habits contraindicate the performance of æsthetic operations, or in those of poor patients who cannot pay for expensive work.

This sweeping condemnation is not borne out by the experience of those who have used it carefully, and has its origin, I think, in the careless, unskilful operations of dentists who inserted it into cavities only half cleaned out. Many of these careless operations, however, preserved teeth for a considerable number of years, and gave the material at the same time both its good and its bad name.

Providing that a cavity is prepared as it should be for a gold filling,—viz., the walls and floor formed of sound, hard dentine (or the floor, if soft dentine is left as pulp protection covered with a white cement),—there will be no discoloration of the tooth unless the walls are so thin that the filling shows through them, and this is not in itself discoloration.

If, however, the decay is not thoroughly removed, or if the dentine is more or less soft, the copper salts will harden this decay or soft dentine and turn it a dark color. The resulting discoloration of the tooth will therefore greatly depend on its structure and on the thoroughness of the excavation.

There appears to be a considerable difference of opinion as to whether copper amalgam shrinks or not. I have never yet seen a case in which this could be noticed by an examination of fillings in the mouth. I have occasionally extracted teeth containing large fillings of either copper or alloy amalgams which were preserving the teeth perfectly as far as they were concerned. In a few of these cases I have split the teeth and removed the fillings intact. I noticed that when the filling was of copper its interior surfaces were of a dark-grayish hue, very similar to the pellets as received for use, the exterior surface being as usual quite black. In alloy fillings, although in some cases the exterior surface was clean and bright, the interior was perfectly black. The cases here alluded to are too few in number to "swear by," but they are instructive as far as they go, the gray hue of the copper proving that no moisture had been admitted to cause oxidation, while the blackness of the interior surfaces of the alloy shows, on the contrary, the result of leakage. It is unnecessary to enter into details of why I extracted teeth containing good fillings, but I may mention that it was usually in cases where pulps had died some long time after capping, and circumstances prevented removal of fillings and treatment.

All who write or speak about copper amalgam allude to the surface wasting, but I have not noticed any allusion to what I consider far more serious,—viz., a wasting away at the cervical edge.

This does not appear to occur nearly so frequently as the surface or general wear. In fact, it may be said to be the exception, while the latter is the rule, but, nevertheless, it occurs sufficiently frequently to be very annoying and to make one doubt the policy of placing a layer of copper amalgam at the cervical margin when filling with some other material.

As an illustration I will describe the following case: About three years ago my brother had several teeth filled by one of the staff of the Dental Hospital, London. In the second right lower molar a large copper amalgam filling was inserted. The whole of the mesial surface was filled, and it also extended half into the crown. On the mesial surface it extended to the edge of the gum, but not below it. The first molar had been extracted some time previously, and the space is now undiminished. I examined this filling about a year after it was inserted and found that there was a distinct wasting away at the gum line, forming a trench or gutter at this part. I scraped it out with a spoon excavator and found I could easily remove the soft, disintegrated filling. This soft layer was not of any very great depth, and I found it necessary to drill a few retaining pits before repairing.

The drilling of these pits was accomplished with difficulty owing to the extreme hardness of the main body of the filling. I then filled up the gutter with Sullivan's cement. A year subsequently I found that my patch had wasted away and the gutter seemed deeper than before. I then patched with an alloy amalgam. In another year,—viz., last September,—I found the alloy patch in place, but from the upper edge of it the copper filling had been wasting away at a rapid rate. It was so soft that I scraped the whole of the mesial surface away with a spoon excavator, and also right under the coronal surface (which was hard), and this I easily removed with an upward push. This appears to be quite unconnected with the ordinary or surface wasting. It is worthy of notice that, owing to the absence of the first molar, there was no possibility of food lodging against the surface of this filling or the oral fluids stagnatory there.

I have for some time past been using one of the new American copper amalgams, hoping that this might not occur with a better preparation, but it requires an observation of several years to find this out. I missed a grand opportunity of testing the American preparation by not refilling my brother's tooth with it, but I thought he had already swallowed as much copper as was good for him.

I have come to the conclusion that a copper amalgam filling saves teeth of good structure simply because it makes a good tight plug; that in teeth containing soft dentine it also saves by hardening it and preventing decay taking place *under* the filling, but that it possesses no power whatever to prevent decay from external causes. That is to say, that if decaying influences collect around the margin of a copper filling, they are just as likely to cause decay as if the tooth were well filled with gold; neither more nor less. The progress of the decay may perhaps not be so rapid when once the outer wall is cracked, but he who thinks that the antiseptic properties of copper amalgam will form a zone of protection around the margins of the filling will be speedily undeceived; at least, such is my experience.

There are alloys on the market which are stated to be improved by the addition of a little copper. Why not add a little of something to the copper amalgam with the object of preventing this surface and cervical wasting, without impairing its antiseptic properties or causing it to shrink. Surely this is not beyond the skill of our chemists.

William C. Grayston.

Scarborough, England.

